# DNA Methylation: An Important Biomarker and Therapeutic Target for Gastric Cancer

**DOI:** 10.3389/fgene.2022.823905

**Published:** 2022-03-04

**Authors:** Yunqing Zeng, Huimin Rong, Jianwei Xu, Ruyue Cao, Shuhua Li, Yanjing Gao, Baoquan Cheng, Tao Zhou

**Affiliations:** ^1^ Department of Gastroenterology, Qilu Hospital, Cheeloo College of Medicine, Shandong University, Jinan, China; ^2^ Department of Reconstructive Surgery, Qilu Hospital, Cheeloo College of Medicine, Shandong University, Jinan, China; ^3^ Department of Pancreatic Surgery, Qilu Hospital, Cheeloo College of Medicine, Shandong University, Jinan, China; ^4^ Department of Geriatric Medicine, Qilu Hospital, Cheeloo College of Medicine, Shandong University, Jinan, China

**Keywords:** DNA methylation, gastric cancer, diagnosis, therapy, prognosis

## Abstract

Gastric cancer (GC) is a very common malignancy with a poor prognosis, and its occurrence and development are closely related to epigenetic modifications. Methylation of DNA before or during gastric cancer is an interesting research topic. This article reviews the studies on DNA methylation related to the cause, diagnosis, treatment, and prognosis of gastric cancer and aims to find cancer biomarkers to solve major human health problems.

## 1 Introduction

Gastric cancer (GC) is a major unresolved clinical problem, with over a million new cases globally in 2020 (Sexton et al., 2020). It is the fourth most common cancer in men (Sexton et al., 2020), and the third most frequent cause of cancer-related deaths worldwide (Global, 2019). Most patients are diagnosed at an advanced stage and have a poor prognosis (Digklia and Wagner, 2016). Recently, multiple studies showed that epigenetic dysregulations, including DNA methylation, histone post-translational modifications, chromatin remodeling and non-coding RNAs, play a vital role in the oncogenesis and progression of GC. Among the epigenetic modifications mentioned, DNA methylation is the earliest known and well-investigated epigenetic change (Skvortsova et al., 2019).

The types of aberrant DNA methylation in human cancers include global DNA hypomethylation and local hypermethylation of genes. Genome-wide hypomethylation mainly occurs in repetitive elements that are normally hypermethylated to maintain genomic integrity and stability. Long Interspersed Nucleotide Element 1 (LINE-1), Alu repetitive elements and human endogenous retroviruses (HERVs) are the major constituents of interspersed repetitive sequences (IRS) (Chansangpetch et al., 2018). Regional hypermethylation of genes occurs in CpG (5′-cytosine-phosphate-guanine-3′) islands, which are normally unmethylated and cause silencing of tumor-suppressor genes, cell cycle regulator genes, and DNA repair genes (Eyvazi et al., 2019; Moore et al., 2013; Shao et al., 2018; Zeng et al., 2017). CpG islands (CGI) are frequently found in mammalian promoters (Field et al., 2018). Three types of DNA methyltransferases including DNMT1, DNMT3A, and DNMT3B, are responsible for DNA methylation. DNMT3A and DNMT3B are primarily ab initio methyltransferases, while DNMT1 maintains the methylation of symmetrically methylated CpGs during DNA duplication (Ebrahimi et al., 2020; Zeng et al., 2017).

Although many studies explored the prospect of DNA methylation as a biomarker with the aim of decreasing GC deaths, the methylation levels in those studies were mainly detected from the tissues by invasive methods. There are still many aberrantly methylated genes whose roles in GC have not been fully investigated, especially those detected by non-invasive methods. Novel non-invasive biomarkers are necessary for early detection, the prediction of prognosis and recurrence, and the evaluation of treatment efficacy. In this review, from the perspective of clinical practicality, we briefly described DNA methylation associated with pathogens of GC. Then, we highlighted the value of aberrant DNA methylation as a non-invasive biomarker for GC management.

## 2 DNA Methylation Associated With Pathogenesis

Pathogens invade host cells and cause epigenetic changes, such as DNA methylation, making it a safer environment for the pathogen. This allows the infection to persist and promotes the development of GC (Fattahi et al., 2018). The most important pathogens associated with gastric carcinogenesis are *Helicobacter pylori* (*H. pylori*, HP) and the Epstein-Barr virus (EBV).

### 2.1 Infection Mediated by *Helicobacter pylori*

Infection by *H. pylori* induces hypermethylation in the promoter regions of many DNA repair genes and tumor suppressor genes (MHL1, RUNX 3, APC, and PTEN), thus silencing the genes and facilitating carcinogenesis (Muhammad et al., 2019). Kosumi et al. found that LINE-1 hypomethylation of non-cancerous gastric mucosae in gastric cancer patients was significantly correlated with *H. pylori* infection (*p* = 0.037) and prospectively confirmed the similar result in non-gastric cancer patients (*p* = 0.010) (Kosumi et al., 2015). Yoshida et al. found that compared to the gastric mucosae of *H. pylori*-negative healthy volunteers, the Alu methylation level was significantly lower in the gastric mucosae of *H. pylori*-positive healthy volunteers and *H. pylori*-positive gastric cancer patients (Yoshida et al., 2011).

When chronic inflammation, triggered by *H. pylori* infection in Mongolian gerbils, was repressed by cyclosporin A, aberrant DNA methylation was substantially suppressed; however, the abundance of *H. pylori* in the gastric mucosa was not reduced. Therefore, it was concluded that the inflammation, rather than *H. pylori*, was responsible for inducing abnormal DNA methylation (Niwa et al., 2013). However, recurrent inflammation caused by alcohol or saturated NaCl did not induce abnormal DNA methylation (Hur et al., 2011). *Helicobacter pylori* infection activates the secretion of IL-1β and TNF-α and the production of reactive oxygen species. Together, they induce DNA methyltransferase 1 (DNMT1) and cause aberrant DNA methylation in gastric epithelial cells (Kim, 2019). Aggregation of aberrantly methylated DNA in the gastric mucosa might favor cancerogenesis (Maeda et al., 2017).

Although eliminating *H. pylori* significantly decreases methylation of tumor suppressor genes, DNA methylation does not return to the same level as that in individuals who are never infected by *H. pylori,* and the higher levels of methylated DNA in the previously infected individuals have adverse effects on the gastric mucosa in the long term (Nakajima et al., 2010). Therefore, individuals with ongoing presence of aberrant DNA methylation would face a higher risk of GC even after the eradication of *H. pylori* (Shin et al., 2012). From this perspective, determination of DNA methylation in *H. pylori*-negative subjects, including subjects whose *H. pylori* has been eliminated, can also act as a helpful diagnostic biomarker for assessing the risk of gastric cancer (Tahara and Arisawa, 2015).

### 2.2 EBV Infection

Based on The Cancer Genome Atlas (TCGA) project, gastric cancer was classified into four molecular subtypes: Epstein-Barr virus, microsatellite instability, genomically stable, and chromosomal instability (Sohn et al., 2017). Epstein-Barr virus-associated gastric carcinoma (EBVaGC), which comprises nearly 10% of gastric carcinomas (Fukayama and Ushiku, 2011), shows the most extreme DNA hypermethylation in all human malignancies (Usui et al., 2021). Matsusaka et al. divided GC into three epigenotypes according to DNA methylation patterns: EBV−/low methylation, EBV−/high methylation, and EBV+/high methylation (Matsusaka et al., 2011). CXXC4, TIMP2, and PLXND1 genes are methylated only in the EBV + tumors. COL9A2, EYA1, and ZNF365 genes are methylated both in EBV+ and EBV−/high tumors. AMPH, SORCS3, and AJAP1 genes are methylated in all gastric cancers (Matsusaka et al., 2011). Many studies have shown that promoter hypermethylation plays a crucial role in the carcinogenesis of EBVaGC (Kaneda et al., 2012; Matsusaka et al., 2014; Okada et al., 2013; Yau et al., 2014; Zong and Seto, 2014). EBVaGC exhibited promoter hypermethylation in multiple genes (e.g., p15, p16, p73, hMLH1, MGMT, GSTP1, CDH1, TIMP1, TIMP3, DAPK, bcl-2, APC, PTEN, and RASSF1A) associated with regulation of cell proliferation (Shinozaki-Ushiku et al., 2015). In contrast, the relationship between EBV infection and demethylation in interspersed DNA repeats is unclear.

Unlike HP infection, EBV infection-induced hypermethylation is considered to be caused not by the intermediate pathways related to inflammation induced by infection but by the pathogen itself (Kaneda et al., 2012). The molecular mechanism of aberrant DNA methylation triggered by EBV infection remains unclear. The proposed mechanism includes the upregulation of the expression of DNMT1 and DNMT3b and the downregulation of the activity of TET2 demethylase by EBV (Hino et al., 2009; Namba-Fukuyo et al., 2016; Zhao et al., 2013).

## 3 Diagnostic Biomarkers

**FIGURE 1:**
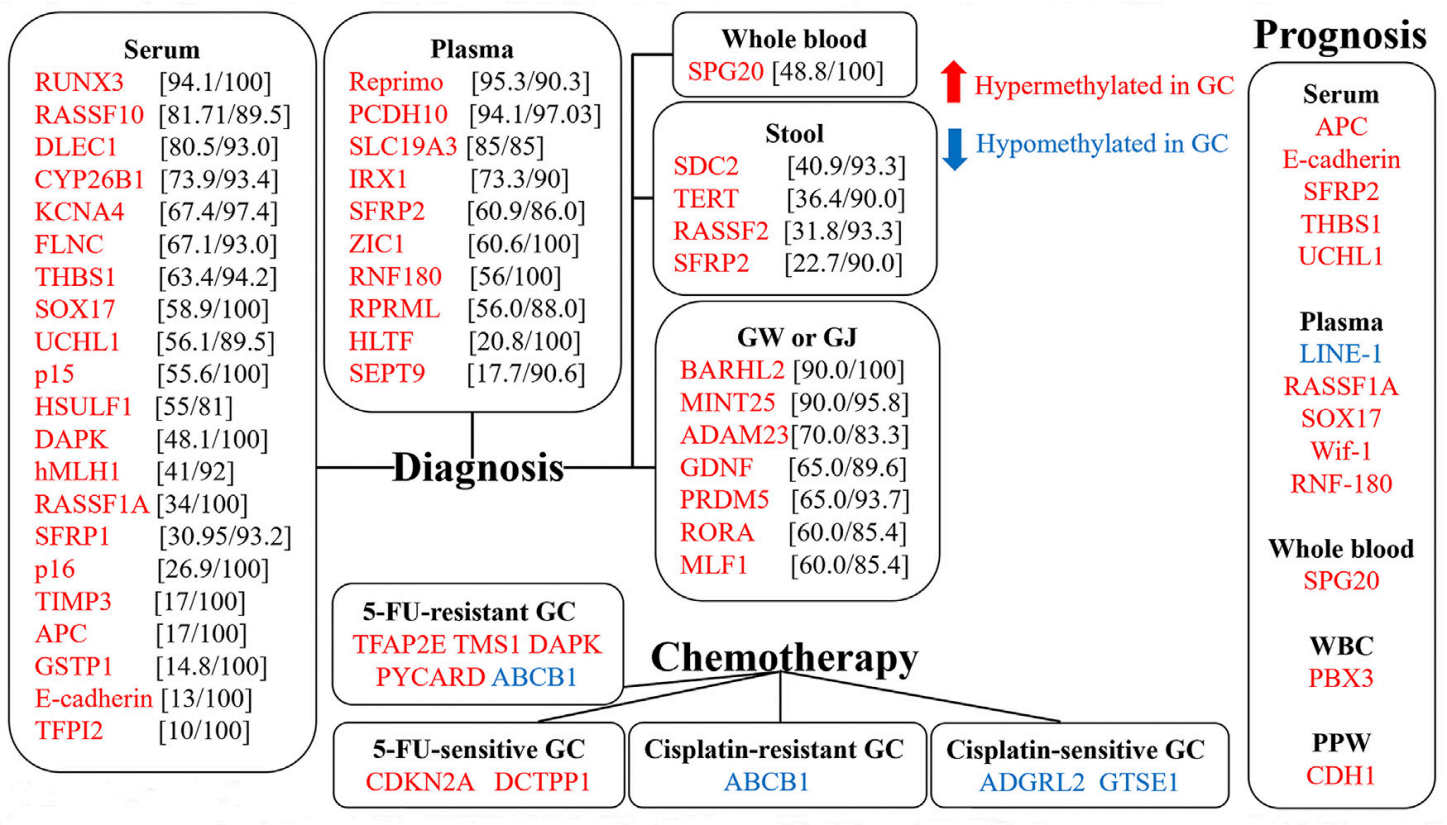
Biomarkers for diagnosis, therapy, and prognosis of gastric cancer. Hypermethylated (in red) and Hypomethylated (in blue) genes are shown. Sensitivity and specificity are shown in square brackets. GW: gastric washes. GJ: gastric juice. WBC: white blood cell. PPW: preoperative peripheral washes.

Exploring the role of DNA methylation in early diagnosis of gastric cancer is important for reducing mortality. Most investigations of DNA methylation are based on the evaluation of disparities in methylation levels between the tumor and the adjacent tissues. Many studies have found that repetitive element hypomethylation and site-specific CpG island promoter hypermethylation are associated with increased risk of GC (Hu X et al., 2021; Min et al., 2017; Saito et al., 2012). In such cases, surgery is required to access the affected tissue, which is a major limitation for clinical application. Growing evidence has shown that methylation-related alterations in cancer patients arise systematically and might be measured in surrogate tissues (Yuasa, 2010). Developing non-invasive detection techniques is quite important for GC patients. Therefore, increasing researchers are devoted to exploring the clinical significance of aberrant DNA methylation detected in body fluids (including peripheral blood, gastric juice, etc.) and stool. Methylation of tumor suppressor genes in peripheral blood has been studied most extensively. In contrast, non-invasive tests related to genome-wide hypomethylation are performed less frequently. Aberrant methylation of multiple genes in body fluids and stool could be a valuable non-invasive biomarker for the early screening and diagnosis of gastric cancer (Qu et al., 2013; Yamamoto et al., 2020) (Table 1) (Figure 1).

**TABLE 1:** Aberrant DNA methylation as diagnostic biomarkers in body fluids and stool of GC patients.

Study	Source	Hypermethylated gene (sensitivity; specificity)	Methods	References
Watanabe, 2009	Gastric wash	MINT25 (90.0%; 95.8%)	Pyrosequencing	Watanabe et al. (2009)
		RORA (60.0%; 85.4%)		
		GDNF (65.0%; 89.6%)		
		ADAM23 (70.0%; 83.3%)		
		PRDM5 (65.0%; 93.7%)		
		MLF1 (60.0%; 85.4%)		
Yamamoto, 2016	Gastric juice-derived exosomes	BARHL2 (90.0%; 100%)	Pyrosequencing	Yamamoto et al. (2016)
Zhang, 2014	Whole blood	SPG20 (48.8%; 100%)	MSP	Zhang et al. (2014)
Liu, 2015	Serum	SFRP1 (30.95%; 93.2%)	MSP	Liu et al. (2015)
Zheng, 2011a	Serum	KCNA4 (67.4%; 97.4%)	Q-MSP	Zheng et al. (2011a)
		CYP26B1 (73.9%; 93.4%)		
Lee, 2002	Serum	p15 (55.6%; 100%)	MSP	Lee et al. (2002)
Kanyama, 2003	Serum	p16 (26%; 100%)	MSP	Kanyama et al. (2003)
Abbaszadegan, 2008	Serum	p16 (26.9%; 100%)	MSP	Abbaszadegan et al. (2008)
Wang, 2008	Serum	RASSF1A (34%; 100%)	MSP	Wang et al. (2008)
Lu, 2012	Serum	RUNX3 (94.1%; 100%)	Q-MSP	Lu et al. (2012)
Chen, 2009	Serum	HSULF1 (55%; 81%)	MSP	Chen et al. (2009)
Hibi, 2011	Serum	TFPI2 (10%; 100%)	Q-MSP	Hibi et al. (2011)
Leung, 2005	Serum	TIMP3 (17%; 100%)	Q-MSP	Leung et al. (2005)
		APC (17%; 100%)		
		E-cadherin (13%; 100%)		
		hMLH1 (41%; 92%)		
Lee, 2002	Serum	E-cadherin (57.4%; 100%)	MSP	Lee et al. (2002)
		DAPK (48.1%; 100%)		
		GSTP1 (14.8%; 100%)		
Xue, 2016	Serum	RASSF10 (81.71%; 89.5%)	BSP	Xue et al. (2016)
Wang, 2015	Serum	FLNC (67.1%; 93.0%)	Q-MSP	Wang et al. (2015)
		THBS1 (63.4%; 94.2%)		
		UCHL1 (56.1%; 89.5%)		
		DLEC1 (80.5%; 93.0%)		
Balgkouranidou, 2013	Serum	SOX17 (58.9%; 100%)	MSP	Balgkouranidou et al. (2013)
Guo, 2011	Plasma	HLTF (20.8%; 100%)	MSP	Guo et al. (2011)
Bernal, 2008	Plasma	Reprimo (95.3%; 90.3%)	MSP	Bernal et al. (2008)
Alarcon, 2020	Plasma	RPRML (56.0%; 88.0%)	MethyLight assay	Alarcon et al. (2020)
Miao, 2020	Plasma	SFRP2 (60.9%; 86.0%)	Q-MSP	Miao et al. (2020)
Cheung, 2012	Plasma	RNF180 (56%; 100%)	Q-MSP	Cheung et al. (2012)
Ng, 2011	Plasma	SLC19A3 (85%; 85%)	Q-MSP	Ng et al. (2011)
Pimson, 2016	Plasma	PCDH10 (94.1%; 97.03%)	MSP	Pimson et al. (2016)
Guo, 2010	Plasma	IRX1 (73.3%; 90%)	MSP	Guo et al. (2010)
Lee, 2013	Plasma	SEPT9 (17.7%; 90.6%)	PCR	Lee et al. (2013)
Chen, 2015b	Plasma	ZIC1 (60.6%; 100%)	MSP	Chen et al. (2015b)
Guo, 2021	Stool	SDC2 (Train set: 40.9%, test set: 40.9%; Train set: 93.3%; test set: 91.7%)	PCR	Guo et al. (2021)
		TERT (Train set: 36.4%, test set: 34.1%; Train set: 90.0%; test set: 91.7%)		
		RASSF2 (Train set: 31.8%; Train set: 93.3%)		
		SFRP2 (Train set: 22.7%; Train set: 90.0%)		
Liu, 2017	Stool	TERT (54.3%; 90%)	PCR	Liu et al. (2017)

Abbreviations: MSP, methylation-specific PCR; q-MSP, Quantitative methylation-specific PCR; BSP, bisulfite sequencing PCR.

### 3.1 Biomarkers in Peripheral Blood

Various blood biomarker-based tests can be used for the early diagnosis of cancer (Huang et al., 2021). Tumor cells can release DNA into peripheral blood, causing abundant circulating DNA levels in the blood of cancer patients to be several-fold higher than that of individuals without cancer (Tahara and Arisawa, 2015). Increasing evidence indicates that the detection of methylated DNA in peripheral blood is more well-developed and stable than detection of gene mutation (Qi et al., 2016). Testing DNA methylation in the blood as a risk marker for carcinoma is of special significance, since the non-invasive and convenient collection of peripheral blood DNA is easily accepted by patients.

#### 3.1.1 Circulating Cell-free DNA Methylation

Circulating cell-free DNA (cfDNA), derived from both normal and tumor cells, is present in the blood. In particular, the cfDNA that is derived from tumors and possesses tumor-specific mutations is called circulating tumor DNA (ctDNA) (Pessoa et al., 2020). Numerous studies have investigated the feasibility of measuring serum or plasma DNA methylation to detect methylation of tumor-derived circulating DNA as a latent diagnostic biomarker for gastric cancer (Tahara and Arisawa, 2015). Methylation of p16, CDH1, RARβ, Reprimo, Rassf1A, hMLH1, RUNX3, APC, E-cadherin, SFRP1, KCNA4, p15, SFRP2, HSULF1, PCDH10, TFPI2, TIMP3, CYP26B1, DAPK, DLEC1, FLNC, THBS1, UCHL1, GSTP1, HLTF, RPRML, SLC19A3, RNF180, etc. are markedly higher in the DNA from peripheral blood of GC subjects than in that of control subjects (Wang et al., 2015; Abbaszadegan et al., 2008; Alarcón et al., 2020; Balgkouranidou et al., 2013; Bernal et al., 2008; Chen et al., 2015a; Chen et al., 2009; Cheng et al., 2007; Cheung et al., 2012; Guo et al., 2010, 2011,; Hibi et al., 2011; Huang et al., 2021; Ikoma et al., 2006; Kanyama et al., 2003; Lee et al., 2002; Leung et al., 2005; Liu et al., 2015; Lu et al., 2012; Miao et al., 2020; Ng et al., 2011; Pimson et al., 2016; Sakakura et al., 2009; Tahara et al., 2013; Wang et al., 2008; Xue et al., 2016; Zhang et al., 2010, 2014; Chen et al., 2015b; Zheng et al., 2011b). Lin et al. evaluated the methylation state of three genes (ZIC1, HOXD10, and RUNX3) from the blood samples of GC patients using methylation-specific polymerase chain reaction. They discovered that the Odds ratios of ZIC1, HOXD10, and RUNX3 methylation for predicting GC were 4.285 (95%CI: 2.435–7.542), 3.133 (95%CI: 1.700–5.775), and 2.674 (95%CI: 1.441–4.960), respectively. The joint detection sensitivity of these three genes was 91.6%. Therefore, the combined detection of multiple gene promoter hypermethylation exhibited a cooperative effect compared to a single biomarker used to predict GC (Lin et al., 2017).

What is noteworthy is that even for the same methylated gene, there are significant discrepancies between the results of different studies, which might be attributed to differences in the sample size, detection methods, and study regions (Huang et al., 2021). To understand this heterogeneity and evaluate the accuracy of DNA methylation markers in the blood for identifying gastric cancer patients, Hu et al. conducted a meta-analysis with 32 studies, containing 69 analyses of blood DNA methylation tests that were conducted to evaluate GC. The 32 studies included 2,098 GC patients and 2,098 control subjects. The blood test based on DNA methylation had an overall sensitivity of 57% and specificity of 97% for gastric cancer. Plasma-based tests showed a sensitivity of 71% and specificity of 89%. Serum-based tests showed a sensitivity of 50% and specificity of 98%. The sensitivity of using multiple methylation genes was 76% and specificity was 85%. These results suggested that the blood-based DNA methylation test has high specificity but moderate sensitivity for the detection of gastric cancer. The determination of various methylation genes or the use of plasma samples might improve the sensitivity of the diagnosis (Hu et al., 2017).

#### 3.1.2 DNA Methylation in Peripheral Blood Leukocytes

Unlike tumor DNA, leukocyte DNA can be obtained non-invasively and relatively inexpensively (Tahara et al., 2018). Studies which determine whether selected tumor suppressor genes and genome-wide repetitive sequence methylation in peripheral blood leukocytes of subjects with gastric cancer and healthy controls are different are rapidly emerging. Hypermethylation of KIBRA, DLEC1, FAT4, WT1, H19, MALAT1, APC, ACIN1, BCL2, CD44, TNFRSF10C and RARB promoters in peripheral blood leukocytes was found to be statistically significant in GC patients (Dauksa et al., 2014; Hu D. et al., 2021; Sun et al., 2018; Xie et al., 2020; Zhang et al., 2018; Zhou et al., 2019). To date, only a few studies have examined GC risk associated with Alu and LINE-1 methylation in peripheral blood leukocytes and the results are variable. Dauksa et al. found that the mean methylation level in Alu and LINE-1 repeats of GC patients was slightly lower than the mean level in the controls (Dauksa et al., 2014). Hou et al. demonstrated that GC risk increased with a decrease in the methylation of Alu or LINE-1, although the trends were not statistically significant (Hou et al., 2010). Gao et al. found that Alu methylation in blood leukocyte DNA was inversely associated with GC risk, but LINE-1 methylation levels were not correlated with GC risk (Gao et al., 2012). Barchitta et al. also showed that the LINE-1 methylation levels were significantly different in tissue samples but not in blood samples (Barchitta et al., 2014). These results suggested that studies with more individuals must be performed to determine the clinical applicability of leukocyte DNA methylation to detect gastric cancer non-invasively.

#### 3.1.3 DNA Methylation in Whole Blood

Several studies evaluated the association of aberrant DNA methylation with the risk of gastric cancer. SOCS3, SPG20, and SFRP1 promoter hypermethylation in whole blood significantly increased GC risk (Han et al., 2020; Tahara et al., 2013; Zhang et al., 2014).

### 3.2 Biomarkers in Gastric Washes

Since a large number of mucosal cells could be extracted from gastric juice (GJ)), DNA biomarkers in gastric juice might be used to detect gastric cancer. However, DNA is easily degraded in an acidic environment; thus, gastric wash (GW) is used as an alternative source for determining aberrant DNA methylation (Yamamoto et al., 2020). Unfortunately, only several early studies showed that the methylation levels of MINT25, RORA, GDNF, ADAM23, PRDM5, CDH1, and MLF1 in gastric washes of GC patients were significantly higher than those of control subjects (Muretto et al., 2008; Watanabe et al., 2009). Among them, MINT25 methylation has the optimal sensitivity (90.0%) and specificity (95.8%), and thus, can distinguish GC from non-GC and be a potential biomarker for screening GC. Yoshiyuki et al. investigated the relationship of the methylation levels between biopsy and gastric washes. The methylation levels of all six genes were tightly associated by statistical analysis (MINT25: *p* = 0.001; RORA: *p* = 0.03; PRDM5: *p* < 0.001; MLF1: *p* < 0.001; ADAM23 *p* < 0.001; GDNF: *p* < 0.001). These results indicated that DNA from gastric washes can be used as a suitable substitute for DNA from biopsied tissues to determine the accumulation of DNA methylation in GC patients (Watanabe et al., 2009).

Hypermethylation of BARHL2 was detected in gastric wash-derived and gastric juice-derived exosomal DNA in early-stage GC patients before endoscopic treatment, whereas methylation levels considerably decreased with a curative endoscopic therapy. These results indicated that BARHL2 methylation might contribute to the detection of residual cancer after endoscopic resection and the potential prediction of tumor relapse (Yamamoto et al., 2020). Some disturbing factors such as aging, HP infection, and chronic inflammation can also induce aberrant DNA methylation. BARHL2 methylation is not affected by those factors. Therefore, GW or GJ exoDNA-based methylation analysis of BARH2 is expected to be an accurate biomarker for detecting early and advanced gastric cancer (Yamamoto et al., 2016).

### 3.3 Biomarkers in Stool

Guo et al. (Guo et al., 2021) evaluated the feasibility of gene methylation in feces for screening gastric cancer. All GC patients and normal controls were divided into training sets and test sets. The sensitivity and specificity of a single marker for gastric cancer detection in the training set for SDC2 were 40.9 and 93.3%, for TERT were 36.4 and 90.0%, for RASSF2 were 31.8 and 93.3%, for SFRP2 were 22.7 and 90.0%, and for Hb were 27.3 and 90.0%. The sensitivity and specificity of the three markers for methylation of SDC2, TERT, and Hb in the test set for gastric cancer detection were 40.9 and 91.7%, 34.1 and 91.7%, and 25.0 and 86.7%, respectively. The results showed that the methylation of SDC2, SFRP2, TERT, and RASSF2 has a certain sensitivity and high specificity in GC screening, which is a potential fecal biomarker of gastric cancer. Another study (Liu et al., 2017) also suggested the feasibility of stool TERT promoter methylation analyses for the non-invasive screening of gastric cancer.

## 4 Therapeutic Target

DNA methylation is also critical for the adjuvant treatment of gastric cancer. Chemotherapy is one of the major methods for treating GC. The main problem with chemotherapy is drug resistance, which is primarily related to DNA methylation. Correcting aberrant methylation patterns can improve chemotherapy response and patient survival (Housman et al., 2014). Animal studies have also shown that direct repression of aberrant DNA methylation can inhibit gastric carcinogenesis (Maeda et al., 2017). Therefore, DNMT inhibitors (DNMTi) are being actively investigated as novel cancer treatments. Additionally, adjuvant radiotherapy of GC has been debated over the past few decades. However, it has been suggested that hypermethylation and inactivation of certain genes associated with cell cycle regulation, DNA repair, apoptosis, and signal transduction can lead to radiotherapy resistance in GC cells (Zhang et al., 2006). In the future, it may be possible to improve radiotherapy response by altering DNA methylation patterns to benefit GC patients.

### 4.1 Chemotherapy

Studies have shown that DNA methylation in gastric cancer cells is related to the sensitivity of chemotherapy and resistance of anticancer agents such as 5-FU and cisplatin. The biomarkers used to identify resistance or sensitivity to chemotherapeutic drugs can be investigated. Hypermethylated TFAP2E, TMS1, PYCARD (ASC/TMS1), and DAPK might be appropriate biomarkers for 5-FU-resistant gastric cancer. Hypermethylated CDKN2A (p16INK4a) and DCTPP1 might be useful biomarkers for 5-FU-sensitive gastric cancer. Hypomethylated ADGRL2 (LPHN2) and GTSE1 are potential biomarkers of cisplatin-sensitive gastric cancer. Regardless of the type of drug, the hypomethylated ATP-binding cassette gene B1 (ABCB1) could be an effective biomarker for chemotherapy-resistant gastric cancer (Choi et al., 2017) (Figure 1). This is because ABCB1 hypermethylation silences genes that encode cellular factors necessary for cancer cell resistance to the chemotherapeutic drugs 5-FU and cisplatin (Shitara et al., 2010).

### 4.2 DNMT Inhibitors

DNMT inhibitors are either nucleoside analogs or non-nucleoside analogs (Erdmann et al., 2015). Azacytidine and decitabine are nucleoside analogs of cytosine that cannot accept a methyl donor at the 5′ position of the pyrimidine ring and depletes cellular DNMT1 (Zeng et al., 2017). Decitabine is integrated into DNA instead of cytidine during duplication, and azacitidine can be incorporated directly into RNA, inhibiting protein synthesis, which causes a substantial reduction in DNMT activity (Navada et al., 2014). Azacitidine was found to suppress the proliferation of GC cell lines and alter DNA methylation (Chen & Wang et al., 2015). Decitabine treatment can cause growth inhibition and reduction in DNMT3A and DNMT3B levels, accompanied by demethylation of the P16 INK4A gene (Liu et al., 2013). Zebularine, another kind of nucleoside analog, is a novel DNMT inhibitor that reduces the expression of the DNMT protein and reactivates epigenetically silenced genes (Tan et al., 2013).

At present, neither azacytidine nor decitabine has been identified as monotherapy for gastric cancer in the clinical setting. This might be because DNMT inhibitors alone cannot reactivate gene expression (Si et al., 2010). However, growing evidence suggests that the combination of DNMT inhibitors and traditional chemotherapy can improve chemosensitization by restoring aberrant epigenetic changes. For example, the combined administration of decitabine and 5-FU showed that TFAP2E is reactively expressed in GC by demethylation and an increase in chemosensitization (Wu F. L. et al., 2015). Additionally, azacytidine upregulates DAPK2, DAPK3, RASSF1, and THBS1 genes which might synergize with chemotherapeutic agents (Tan et al., 2013; Wu F et al., 2015; Zhang et al., 2006). In a clinical trial (Phase 1) (Schneider et al., 2017), researchers investigated pre-treatment with 5-azacitidine as a demethylation reagent in late-stage gastric cancer. They used 5-azacytidine (V) before EOX (epirubicin, oxaliplatin, capecitabine) neoadjuvant chemotherapy in GC patients and the result showed hypomethylation of tumor-related loci such as HPP1, TIMP3, CDKN2A, ESR1, and MGMT. Most patients easily tolerate neoadjuvant VEOX therapy with significant clinical and epigenetic responses. More randomized studies are required to further determine whether the efficacy of this combination is better than chemotherapy alone.

Other types of non-nucleoside analogs such as procaine, hydrazone-gallate, genistein, miRNA-21, miRNA-335, miRNA-148a, and miRNA-155-5p have also been investigated, but further studies need to be performed before approval for clinical use (Erdmann et al., 2016; Li et al., 2014, 2018,; Pan et al., 2010; Xie et al., 2014; Zhang et al., 2015; Zuo et al., 2013).

### 4.3 Radiotherapy

Despite active anticancer treatment, the overall prognosis of advanced GC is not ideal. Hence, an effective biomarker is required for selecting suitable patients who might benefit from adjuvant radiotherapy. Unfortunately, such predictive indicators have not been determined.

An et al. (An et al., 2020) analyzed methylation maps of 397 gastric cancer samples downloaded from The Cancer Genome Atlas (TCGA) and established a new biomarker called promoter methylation burden of DNA repair genes (RPMB), which meant the ratio of methylated DNA repair genes to the number of all DNA repair genes in order to identify patients who were sensitive to radiotherapy. Subgroup analyses based on overall survival (OS) and disease-free survival (DFS) showed that most of the subgroups tended toward the high-RMPB group. High-RPMB patients receiving radiotherapy with both ≥ T2 tumor and positive lymph nodes showed longer DFS than the low -RPMB group (*p* = 0.010). High-RPMB patients receiving radiotherapy with both ≥ T2 tumor and positive lymph nodes survived low-RPMB patients in disease-free status (*p* = 0.010). Therefore, RPMB might be a promising biomarker to evaluate the indications for adjuvant radiotherapy in GC. Furthermore, treatment with 5-aza-CdR can positively affect radiotherapy sensitivity of gastric cancer cells by enhancing the expression of some genes such as p53, RASSF1, and DAPK (Qiu et al., 2009).

## 5 Prognostic Biomarkers

Aberrant DNA methylation in peripheral blood is also related to multiple prognostic results of gastric cancer. Therefore, it could be used as a prognostic biomarker of GC (Figure 1). Hypermethylation of most genes such as APC, E-cadherin, UCHL1, SPG20, RASSF1A, SFRP2, CDH1, THBS1, SOX17, Wif-1, RNF-180, MED12L, HMLH1, MGMT, FLNC, LOX, HOXD10, BNIP3, and PCDH10 was significantly associated with adverse prognosis in GC (Balgkouranidou et al., 2015; Cheung et al., 2012; Hu X et al., 2021; Ikoma et al., 2006; Karamitrousis et al., 2021; Necula et al., 2019; Pimson et al., 2016; Wang et al., 2015; Yan et al., 2021; Yu et al., 2012; Zhang et al., 2014) (Table 2). Additionally, Xie et al. found that PBX3 methylation in peripheral blood leukocytes was associated with poorer GC prognosis only in the elderly group (HR = 1.678, 95% CI = 1.046–2.693) and the female group (HR = 2.058, 95% CI = 1.024–4.137) (Xie et al., 2020).

**TABLE 2:** Aberrantly methylated genes as prognostic biomarkers in GC patients.

Study	Source	Aberrantly methylated gene	Prognosis	Methods	References
Balgkouranidou, 2015	Serum	APC	Poorer OS (HR = 4.6, 95% CI = 1.1–20.3, *p* = 0.046)	MSP	Balgkouranidou et al. (2015)
Ikoma, 2006	Serum	E-cadherin	Poorer 3-year survival rate (*p* < 0.05)	MSP	Ikoma et al. (2006)
Yan, 2021	Serum	SFRP2	Shorter PFS (HR = 13.05; 95% CI = 3.05–55.95) and OS (HR = 7.80; 95% CI = 1.81–33.60) (stage III); Shorter PFS (HR = 2.74; 95% CI = 1.58–4.78) and OS (HR = 3.14; 95% CI = 1.67–5.92) (stage IV)	PCR	Yan et al. (2021)
Hu, 2021	Serum	THBS1	Worse DFS (HR = 3.838; 95% CI = 1.691–8.710; *p* = 0.001)	Q-MSP	Hu X et al. (2021)
Wang, 2015	Serum	UCHL1	Worse OS (*p* = 0.033)	Q-MSP	Wang et al. (2015)
		THBS1	Worse OS (*p* = 0.0483)		
Ko, 2021	Plasma	LINE-1	Worse OS (*p* = 0.006)	qPCR	Ko et al. (2021)
Pimson, 2016	Plasma	RASSF1A	Lower OS (HR = 2.33, 95% CI = 1.14–4.85, *p* = 0.002)	MSP	Pimson et al. (2016)
Karamitrousis, 2021	Plasma	SOX17	Lower PFS and OS (*p* < 0.001)	MSP	Karamitrousis et al. (2021)
		Wif-1	Lower PFS and OS (*p* = 0.001)		
		RASSF1A	Lower PFS and OS (*p* = 0.004)		
Cheung, 2012	Plasma	RNF-180	Poorer OS (HR = 2.13; 95% CI = 1.11–4.08; *p* = 0.02)	Q-MSP	Cheung et al. (2012)
Zhang, 2014	Whole blood	SPG20	Shorter OS (*p* = 0.037)	MSP	Zhang et al. (2014)
Xie, 2020	Peripheral blood leukocytes	PBX3	Poorer cum survival (HR = 1.678, 95% CI = 1.046–2.693) (elderly group); Poorer cum survival (HR = 2.058, 95% CI = 1.024–4.137) (female group)	MS-HRM	Xie et al. (2020)
Yu, 2012	PPW	CDH1	Worse DFS (*p* < 0.000)	MSP	Yu et al. (2012)

Abbreviations: CI, confidence interval; HR, hazard ratio; OR, odds ratio; MS-HRM, methylation-sensitive high-resolution melting; PFI, progression-free interval; PFS, progression free survival; PPW, preoperative peritoneal washes.

Ko et al. evaluated the prognostic value of LINE-1 methylation level in cfDNA in gastric cancer patients undergoing radical surgery and chemotherapy. The overall survival (OS) of patients with low methylation levels before starting treatment was significantly worse than those with high methylation levels. But methylation level before surgery had no effect on recurrence-free survival (RFS) and OS (Ko et al., 2021). However, another study showed that the methylation status of LINE-1 in leukocyte DNA was not an independent prognostic factor of GC (Tahara et al., 2018).

The association between aberrant DNA methylation and the prognosis of GC needs to be evaluated in larger cohorts and diverse populations. Additionally, more intensive studies are required to determine the potential molecular biomarkers for predicting prognosis in GC patients.

## 6 Conclusion

*Helicobacter pylori* and EBV are the most important pathogens associated with gastric cancer, which can cause carcinogenesis by inducing aberrant DNA methylation. DNA methylation has high clinical application value. Aberrant methylation of various genes in body fluids and feces can be used as a non-invasive method for early screening and diagnosis of gastric cancer. Specifically, Reprimo, RUNX3, PCDH10, BARHL2, and MINT25 hypermethylation have both high sensitivity and specificity, which indicates their value in the diagnosis of GC. However, the sensitivity of detecting other types of DNA methylation from peripheral blood and stool is not satisfactory. Using a combination of multiple genes can yield higher sensitivity. DNA methylation can also affect the response to chemoradiotherapy in gastric cancer patients. A combination of DNMT inhibitors and chemotherapy drugs seems to have a better therapeutic effect. Therefore, more DNMT inhibitors that have lower toxicity, an effective response, and a low price need to be developed. Furthermore, DNA methylation can predict a variety of prognostic results for GC patients, such as overall survival (OS) and disease-free survival (DFS). Aberrant methylation of APC, SFRP2, LINE-1, E-cadherin, SOX17, Wif-1, RASSF1A, RNF-180, UCHL1, and SPG20 in peripheral blood was significantly associated with shorter OS in GC. The methylation levels of SFRP2, SOX17, Wif-1, and RASSF1A in peripheral blood had an impact on progression-free survival (PFS). THBS1 methylation in the serum and CDH1 methylation in preoperative peripheral washes (PPW) were related to worse DFS. Additionally, aberrantly methylated genes such as SFRP2, THBS1, UCHL1, SOX17, APC, E-cadherin, RASSF1A, RNF-180, and SPG20 could play an important role in both diagnosis and prognosis of GC (Table 3). New tests with improved sensitivity, simplicity, standardization, and cost-effectiveness need to be developed, and new biomarkers need to be validated in larger prospective clinical studies.

**TABLE 3:** Aberrantly methylated genes that play an important role in both diagnosis and prognosis of GC.

SFRP2	
Detection	Sensitivity: 60.9%; Specificity: 86.0% (plasma) Miao et al. (2020)
	Sensitivity: 22.7%; Specificity: 90.0% (stool) Guo et al. (2021)
Prognosis	Shorter PFS (HR = 13.05; 95% CI = 3.05–55.95) and OS (HR = 7.80; 95% CI = 1.81–33.60) (stage III); Shorter PFS (HR = 2.74; 95% CI = 1.58–4.78) and OS (HR = 3.14; 95% CI = 1.67–5.92) (stage IV) (serum) Yan et al. (2021)
THBS1	
Detection	Sensitivity: 63.4%; Specificity: 94.2% (serum) Wang et al. (2015)
Prognosis	Worse DFS (HR = 3.838; 95% CI = 1.691–8.710; *p* = 0.001) (serum) Hu X. et al. (2021)
	Worse OS survival (*p* = 0.0483) (serum) Wang et al. (2015)
UCHL1	
Detection	Sensitivity: 56.1%; Specificity: 89.5% (serum) Wang et al. (2015)
Prognosis	Worse OS (*p* = 0.033) (serum) Wang et al. (2015)
SOX17	
Detection	Sensitivity: 58.9%; Specificity: 100% (serum) Balgkouranidou et al. (2013)
Prognosis	Lower PFS and OS (*p* < 0.001) (plasma) Karamitrousis et al. (2021)
APC	
Detection	Sensitivity: 17%; Specificity: 100% (serum) Leung et al. (2005)
Prognosis	Poorer OS (HR = 4.6, 95% CI = 1.1–20.3, *p* = 0.046) (serum) Balgkouranidou et al. (2015)
E-cadherin	
Detection	Sensitivity: 13%; Specificity: 100% (serum) Leung et al. (2005)
	Sensitivity: 57.4%; Specificity: 100% (serum) Lee et al. (2002)
Prognosis	Poorer 3-years survival rate (*p* < 0.05) (serum) Ikoma et al. (2006)
RASSF1A	
Detection	Sensitivity: 34%; Specificity: 100% (serum) Wang et al. (2008)
Prognosis	Lower OS (HR = 2.33, 95% CI = 1.14–4.85, *p* = 0.002) (plasma) Pimson et al. (2016)
	Lower PFS and OS (*p* = 0.004) (plasma) Karamitrousis et al. (2021)
RNF-180	
Detection	Sensitivity: 56%; Specificity: 100% (plasma) Cheung et al. (2012)
Prognosis	Poorer OS (HR = 2.13; 95% CI = 1.11–4.08; *p* = 0.02) (plasma) Cheung et al. (2012)
SPG20	
Detection	Sensitivity: 48.8%; Specificity: 100% (whole blood) Zhang et al. (2014)
Prognosis	Shorter OS (*p* = 0.037) (whole blood) Zhang et al. (2014)
